# Polymerization of Potassium Azide in Liquid Nitrogen Using Nanosecond-Pulsed Spark Plasma

**DOI:** 10.3390/ma17194787

**Published:** 2024-09-29

**Authors:** Zhiheng Song, Alexander Fridman, Danil Dobrynin

**Affiliations:** C&J Nyheim Plasma Institute, Drexel University, 200 Federal Street, Suite 500, Camden, NJ 08103, USA; zs344@drexel.edu (Z.S.); af55@drexel.edu (A.F.)

**Keywords:** nanosecond-pulsed discharge, polymeric nitrogen, in-liquid discharge

## Abstract

In this manuscript, we report on the synthesis of a polynitrogen material from a potassium azide precursor using nanosecond-pulsed spark discharge plasma in liquid nitrogen. The polynitrogen material was characterized using Raman and Fourier transform infrared (FTIR) spectroscopy and identified as K_2_N_6_, with planar N_6_ rings and K- ions that have P6/mmm symmetry. An analysis of the mechanism behind such a transformation shows the importance of direct plasma–chemical effects in polymerization, while the crystal structure changes are believed to be due to plasma-emitted radiation in the ultraviolet range.

## 1. Introduction

Nitrogen compounds like nitrogen heterocyclic compounds (azine compounds [1], azole compounds [2], and furazan compounds [3]), nitrogen clusters [4], and excited-state nitrogen compounds [5] are examples of high-energy-density materials that are widely used as explosives based on their high ratio of potential chemical energy output to density [6]. Such high-energy-density materials have broad applications, including explosives, pure nitrogen sources, and photographic materials. 

The high energy density of nitrogen compounds is due to the average bond energy difference between the nitrogen single, double, and triple bonds. Nitrogen triple bonds are the most stable atomic bonds and have an average bond energy of 946 kJ/mol. The average bond energy of nitrogen triple bonds is more than double the average bond energy of nitrogen double bonds (419 kJ/mol) and is six times the average bond energy of nitrogen single bonds (159 kJ/mol). Because of the energy difference between the average bond energy, the formation of stable triple-bonded nitrogen from crystalline single-bonded nitrogen would release about 800 kJ/mol energy.

In 1985, McMahan from the Lawrence Livermore National Laboratory (LLNL) and Lesar from the Los Alamos National Laboratory (LANL) predicted that, under high temperatures and high pressures, nitrogen triple bonds would dissociate into nitrogen double or single bonds and that atomic nitrogen would polymerize into a crystalline polymeric nitrogen structure [7]. In 1992, Mailhiot et al. [8] discovered a high-energy and stable polymeric nitrogen with a space group that has an I/213 special gauche cubic structure, defined as cubic gauche nitrogen (cg-N), where eight nitrogen atoms in the unit cell are connected via single bonds. They also predicted the possibility of its synthesis under atmospheric conditions. In 2002, Eremets et al. [9] successfully synthesized the predicted cubic gauche nitrogen by laser heating a diamond anvil cell, where laser heating was applied to nitrogen at a very high pressure (110 GPa) and temperature (up to 2000 K). Such polynitrogen compounds are of interest in the field of high-energy materials, where they could serve as powerful propellants, explosives, or in other energy storage applications due to their high energy density. Additionally, their unique chemical properties might be leveraged in specialized chemical reactions and in the synthesis of novel materials with tailored electronic or mechanical properties. These materials could also find applications in other fields requiring stable, high-energy nitrogen compounds, such as in specialized chemical synthesis and advanced materials science.

Electrical discharges are widely used in the generation of nanostructured materials [10]. Nanosecond-pulsed spark discharge plasma is a potential approach for unconventional high-energy material generation based on its unique properties [11], including high temperature, high pressure, radiation (covering the ultraviolet, visible light, and infrared ranges), highly concentrated active species, and high electric field intensity. The low-temperature environment and fast quenching characteristics of liquid nitrogen fulfil the need for specialized storage and handling equipment, while also offering more opportunities for the stabilization of high-energy materials [12]. The need for high-voltage equipment, a liquid nitrogen source, and the potential costs associated with scaling up the process for industrial applications remain significant challenges. These factors are crucial considerations in evaluating the commercial potential of this technology.

Alkali metal azides such as sodium azides are commonly used as precursors for the generation of polynitrogen materials, based on their behaviors under extremely high-pressure conditions [13]. An azide ion is a straight chain with three nitrogen atoms that are connected via covalent bonds and have weak interactions with each other. The application of a high pressure would decrease the distance between each nitrogen atom and increase the interactions between them. This increase in interaction would lead to a transformation into a larger nitrogen cluster and, further, a polymeric nitrogen network. High-pressure phase transitions have also been reported in rubidium azide (RbN_3_), cesium azide (CsN_3_), and thallium azide (TlN_3_), with relatively low transition pressures (<1 GPa) [14]. Additionally, compression leads to structural phase transitions involving lattice distortions with a direction change or polymerization of the linear azide anions [15]. 

Alkali metals can also stabilize charges that are left over after the polymeric nitrogen material’s formation [16]. In our previous work [17], sodium azide was used as a precursor for the generation of high-energy-density polynitrogen N_6_ materials. Unlike sodium azide, which has C2/m symmetry, potassium azide has I4/mcm symmetry at room temperature. With different precursor crystal structures, the synthesis of different polynitrogen materials with different structures becomes possible. The arrangement and bonding of nitrogen atoms in the precursor can dictate the final configuration and properties of the resulting polynitrogen compound. Different precursor structures provide varying degrees of stability, bonding configurations, and pathways for nitrogen polymerization. However, the need for precise control over the plasma conditions to ensure consistent product formation, the difficulties in stabilizing the high-energy polynitrogen compounds, and the challenges posed by the potential formation of by-products during the synthesis process are significant barriers in the context of achieving scalable and reliable production methods for such materials.

In this study, we report on the nanosecond-pulsed spark discharge treatment of potassium azide in liquid nitrogen and the characterization of the product. We show that plasma treatment results in the generation of a new compound, identified as K_2_N_6_, with planar N_6_ rings that have P6/mmm crystal symmetry. The potential of polynitrogen materials to revolutionize the field of high-energy materials is due to their significantly higher energy densities than current materials. Understanding the plasma-chemical processes that enable the formation of such materials could lead to breakthroughs in other areas of plasma physics and materials science.

## 2. Materials and Methods

**Figure 1:**
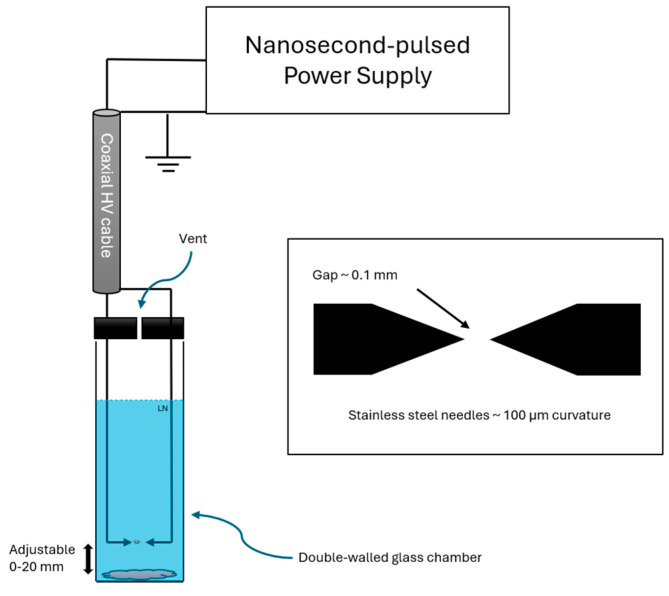
Schematic of experimental setup for generation of nanosecond-pulsed spark plasma in liquid nitrogen.

The experiments were conducted as follows: two stainless steel pin electrodes with 100 μm tip curvature were fixed at a distance of ~0.1 mm for the generation of spark discharge inside a 450 mL double-walled glass flask. Potassium azide powder (Sigma-Aldrich, St. Louis, MO, USA, ≥99% metal basis) was placed at the bottom of the flask. The distance from the discharge to the potassium azide powder was adjustable (Figure 1).

Nanosecond pulses were generated using a FPG 20-05NM high-voltage power supply (FID Technology, Burbach, Germany), which provided pulses with 10.5 kV amplitude for a duration of 10 ns at 90% amplitude and 700 Hz repetition frequency. High-voltage pulses were delivered to the needle electrodes via a 6 m long RG393/U 50 Ω coaxial cable. 

Fourier transform infrared spectroscopy (FTIR) measurements were carried out using a Nicolet 8700 FTIR spectrometer. Raman spectra were measured using a Princeton Instruments-Acton Research TriVista TR555 spectrometer system together with a SDM532-100SM-L Newport 532 nm Spectrum Stabilized Laser Module and RPB532 Raman probe (InPhotonics, Norwood, MA, USA). 

Shadow imaging of the spark discharge was performed using a 4Picos intensified charge coupled device (ICCD) camera (Stanford Computer Optics, Berkeley, CA, USA) with a 75W Xe arc lamp (6251NS, Newport, RA, USA) as the source of the backlight. The emission from the spark discharge was captured using an optical fiber and directed through a Princeton Instruments SP-2500i monochromator, which was configured in the single stage with a 900 lines/mm grating. To correct the spectrum to within the 300–800 nm range, a calibrated quartz tungsten halogen lamp (63 350, Newport, RA, USA) was employed, accounting for camera sensitivity, transmission through optical components, and absorption by liquid nitrogen. Both the imaging and emission spectra were recorded from the discharge operating at a frequency of 10 Hz, synchronized using an AFG-3252 function generator (Tektronix, Beaverton, OR, USA).

## 3. Results and Discussions

### 3.1. Characterization of Spark Discharge in Liquid Nitrogen

**Figure 2:**
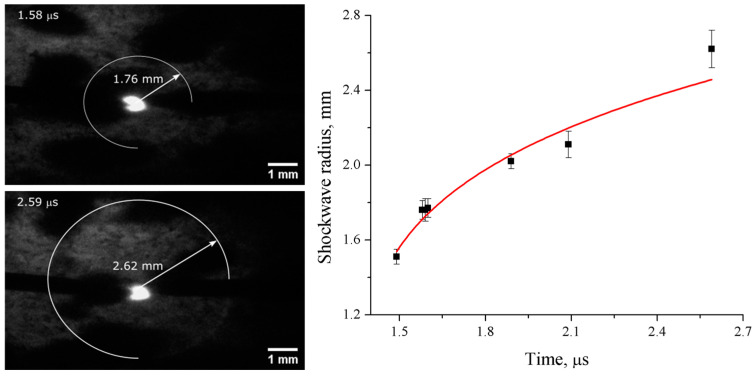
Shadow images of the post-discharge events in liquid nitrogen and the shockwave radii.

Shadow imaging and optical emission spectroscopy measurements of the nanosecond spark plasma in liquid nitrogen were performed on the discharge operating at 10 Hz repetition frequency. Figure 2 shows the shadow imaging of the spark discharge in liquid nitrogen, as well as the radius of the observed shockwave. 

The change in diameter of the shockwave in the shadow imaging measurements shown in Figure 2 was converted into the shockwave propagation velocity. The initial velocity was estimated to be about 3.3 km/s, while later, it decreased to around 0.85 km/s, which corresponds to the speed of sound in liquid nitrogen. Similar shockwave behavior was observed in [18] for nanosecond-pulsed non-thermal corona discharge in liquid nitrogen. The initial velocity was then used to derive the shockwave’s initial pressure using the Rankine–Hugoniot condition [19]:

Conservation of mass:

$$\rho_{1}u_{1}{=\rho_{2}u}_{2}$$

Conservation of momentum:

$$P_{1}+\rho_{1}{u_{1}}^{2}=P_{2}+\rho_{2}{u_{2}}^{2}$$

where the fluid properties before of the shock (1) and after the shock (2) are related by the Rankine–Hugoniot conditions, with *ρ* being the density of the fluid, *u* being the velocity, and *P* being the pressure of the fluid. The post-shock density $\rho_{2}$ can be calculated using the Rankine–Hugoniot relation for density [20]:

$$\frac{\rho_{2}}{\rho_{1}}=\frac{(\gamma +1)M_{1}^{2}}{\left(\gamma -1\right)M_{1}^{2}+2}$$

where γ is the specific heat ratio and *M*_1_ is the Mach number, which can be estimated from the measured velocity as being 3.88. The density ratio was calculated to be about 4.5, and the shockwave pressure was calculated to be about 7 GPa, which corresponds to the result from previous studies on microsecond pulsed discharge in liquid [18,21].

**Figure 3:**
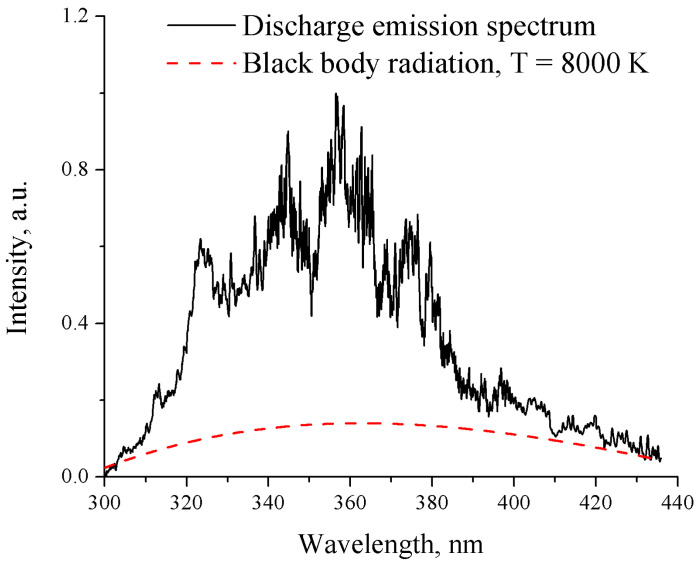
Fit of the optical emission spectrum for nanosecond-pulsed spark discharge in liquid nitrogen to that of black body radiation.

The optical emission spectrum of the spark discharge in liquid nitrogen was measured with a 5 µs exposure time and 20 accumulations. A quartz tungsten halogen-calibrated source lamp (63 350, Newport) was used to calibrate the measurements due to the camera sensitivity difference, transmission through optical components, and absorption by liquid nitrogen. The measured spectrum, shown in Figure 3, was corrected using the calibration function. With a relatively long exposure time (compared with the pulse duration), we were able to record the emission from the spark discharge, including multiple re-ignitions due to pulse reflection. Electrode erosion was also observed after 30 min of treatment, suggesting that thermal effects played an important role in the spark discharge.

To estimate the temperature of the discharge, we considered the shape of the emission spectrum as black body radiation. Fitting the spark discharge emission spectrum to that of black body radiation, as shown in Figure 3, yielded an estimated temperature of approximately 8000 K, which aligns with previous works [22] on nanosecond-pulsed spark discharge in liquid nitrogen. The shadow imaging and optical emission spectroscopy of the nanosecond-pulsed discharge in liquid nitrogen indicate that the discharge region produces both high pressures and temperatures, which will be discussed further in the following sections.

### 3.2. Treatment of Potassium Azide with Nanosecond-Pulsed Plasma in Liquid Nitrogen—Color Change

Approximately 1 g of potassium azide powder was treated for a duration of 30 min with different powder-to-discharge distances, within the range of 0 to 30 mm, with the distance of 0 corresponding to direct contact between the discharge and the precursor. The color of the potassium azide powder changed from white to lilac after the plasma treatment for distances less than 20 mm, and the shorter the distance, the more obvious the observed color change. Treatment at distances of 20 mm or greater resulted in the potassium azide becoming gray. The potassium azide powder was also stored in liquid nitrogen for at least 30 min; no color change was observed. Unlike previous works [23,24], the powder did not explode upon heating.

The color change of potassium azide from white to lilac suggests electronical structure changes induced by plasma treatment. The lilac color, in particular, was reported to be due to color center formation in potassium chloride [25]. A color center (F-center, or Farbe center) is a type of crystal defect where unpaired electrons take over the position of anionic vacancies [26]. The electrons absorb light in the visible region and turn the material from transparent to colored [27]. The color resulting from a color center can be used to identify salts, as it varies for different metal cations [28]. Ionizing radiation is a resulting factor for color centers [29], and plasma-produced UV radiation [24] could cause the formation of a color center in our case. As noticed, a shorter powder-to-discharge distance causes significant color change, and because the intensity of UV radiation is proportional to the reciprocal of distance squared [30], we can conclude that it is indeed UV-induced.

### 3.3. FTIR Spectrum

**Figure 4:**
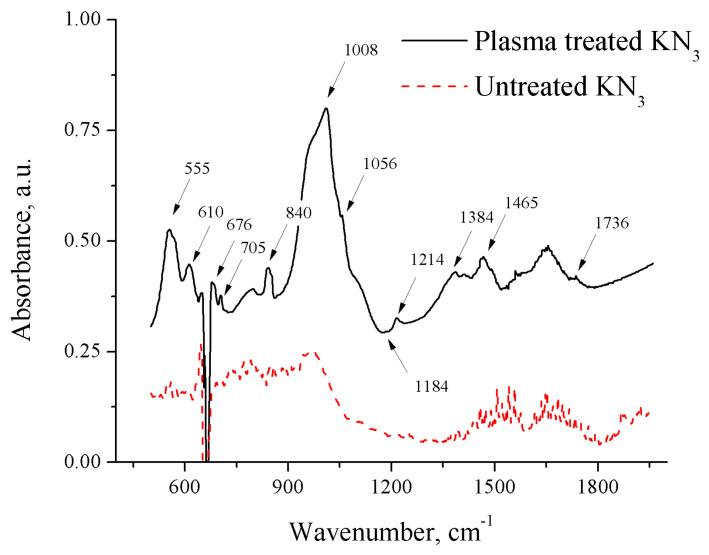
FTIR spectra of potassium azide before and after plasma treatment (spectra shifted vertically for clarity).

The FTIR spectra of the untreated and treated potassium azide are shown in Figure 4. The peaks detected in the untreated powder spectrum correspond to active potassium azide IR peaks [31]. New peaks located at 555, cm^−1^, 610 cm^−1^, 676 cm^−1^, 705 cm^−1^, 840 cm^−1^, 1008 cm^−1^, 1056 cm^−1^, 1184 cm^−1^, 1214 cm^−1^, 1384 cm^−1^, 1465 cm^−1^, and 1736 cm^−1^ were observed after 30 min of spark discharge treatment for potassium azide in liquid nitrogen. No new peaks were obtained at a discharge-to-powder distance of 20 mm or greater. To assign this new set of peaks, we compared them with several density functional theory (DFT) calculations and predicted the high-pressure product vibrational modes listed in Table 1 [32]. The calculations predicted that the most stable structure for the product is D2 hexaazabenzene.

The obtained FTIR spectrum showed good correspondence with the predicted structure, indicating that one of the products obtained via the nanosecond-pulsed spark discharge plasma treatment of potassium azide under liquid nitrogen can be identified as K_2_N_6_ with P6/mmm symmetry and planar D2 N_6_ hexaazabenzene rings located at the corner of the unit cell. Additional peaks can be assigned to other polynitrogen materials or a mixture of multiple structures [33], including the following:

Z-shaped planar N_6_ with two N_3_ fragments connected by a single bond with predicted vibrational frequencies of 690 cm^−1^ and 1529 cm^−1^

Cyclic boat form of N_6_ with dihedral angles of 25.2° and 17.2° with predicted vibrational frequencies of 913 cm^−1^ and 1746 cm^−1^.

Neutral or single negatively charged N_6_ would also be formed in the process, leaving the vacancies to be occupied by electrons, hence inducing a color change.

### 3.4. Raman Spectrum

**Figure 5:**
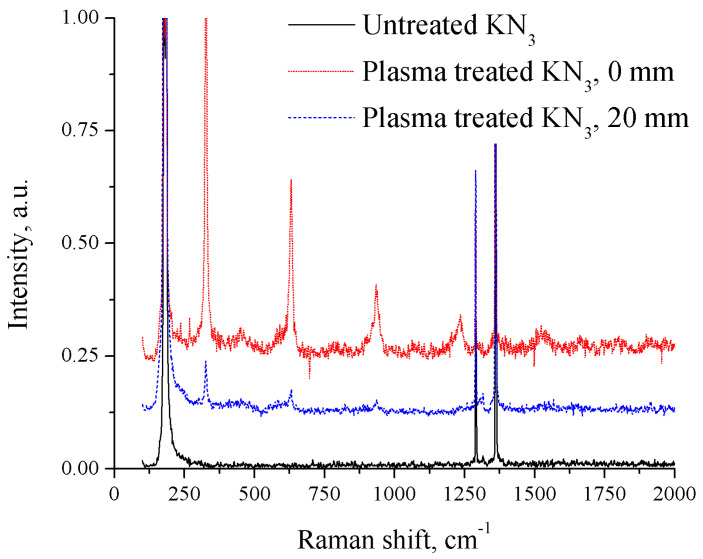
Raman spectra of potassium azide before and after plasma treatment (spectra shifted vertically for clarity).

The Raman spectra of both untreated and plasma-treated potassium azide are shown in Figure 5. In the 20 mm distance setup, several new peaks formed after 30 min of treatment under liquid nitrogen, at 326 cm^−1^, 630 cm^−1^, and 937 cm^−1^. For a shorter-distance setup, two additional peaks located at 1235 cm^−1^ and 1523 cm^−1^ appeared.

Potassium azide has a crystal structure with I4/mcm symmetry at ambient conditions, Raman peaks located at 147 cm^−1^, 1268 cm^−1^, and 1340 cm^−1^ corresponding to the symmetric stretch R, a first overtone of the asymmetric stretch v_1_, and a second overtone of the bending mode 2v_2_ associated with the vibration of potassium azide [34]. The position of the strongest new peak, 327 cm^−1^, corresponds to the high-pressure potassium azide product appearing at about 24.2 GPa with a peak wavelength at around 320 cm^−1^ [35], assigned to the R(E_g_) and R(B_1g_) crystal vibrational modes. As noticed, the set of new peaks has nearly the same shift offset, which can be assigned as the overtone or Fermi resonance of the crystal liberational mode. The predicted pressure of such polymorphs of KN_3_–K_2_N_6_ with P6/mmm symmetry was about 40 GPa [36,37,38] and was obtained experimentally [39] at 49–53 GPa with 2500 K laser heating. Laser heating was used to overcome the large kinetic barrier for such a transformation as the system changes the chemical state. However, the authors of [39] also showed that they were able to trace the p6/mmm phase down to approximately 20 GPa. As estimated in the previous section, the nanosecond-pulsed spark discharge would result in gigapascal pressure, which provided the high-pressure condition for polymerization. Additionally, the predicted intensity of the Raman peaks for planar N_6_ is relatively low [32], so some of the predicted peaks would not be obtained by our system due to the weak Raman intensity and the detection limit. Together with FTIR DFT prediction, the product of nanosecond-pulsed spark discharge treatment of potassium azide is in good agreement with the predicted K_2_N_6_, with planar N_6_ rings and K-ions having P6/mmm symmetry.

### 3.5. Mechanism Study of Color Change, Crystal Structure Change, and Polymerization

In this study, we considered several factors that could result in the observed color change, crystal structure alteration, and polymerization of potassium azide. A Raman spectrum analysis indicated that the crystal structure’s transformation was minimally affected by varying the distance between the discharge source and the powder. This suggests that relatively stable active species might play a significant role. Additionally, UV radiation produced by the nanosecond-pulsed spark discharge was identified as a potential contributing factor.

We constructed various experimental arrangements within a glass chamber, all set at a consistent discharge-to-powder distance of 10 mm but differing in their shielding effects (Table 2):

1. Covered with a quartz window, allowing certain wavelengths of UV, visible light, and IR, as well as electric fields to pass through but blocking the direct effects of plasma-generated reactive species;
2. Enclosed with a metal mesh, which, being a conductor, nullifies the electric field within it while allowing delivery of all other factors;
3. Shielded by both quartz and metal mesh, eliminating the effects of reactive species and electric fields;
4. Covered with a glass window, eliminating the effects of UV and reactive species.

**Figure 6:**
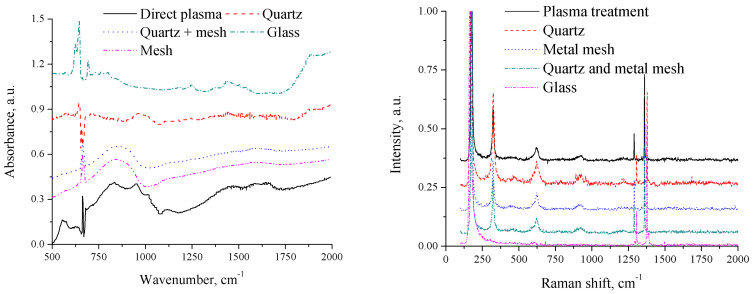
FTIR (**left**) and Raman (**right**) spectra of potassium azide treated by plasma in various setups as described in Table 2 (spectra shifted vertically for clarity).

We assessed color changes visually, polymerization based on the emergence of new peaks in the FTIR spectrum, and crystal structure modifications based on the appearance of a Raman peak at 326 cm^−1^ (Figure 6). The color change in the azide powder to lilac was observed in treatment setups 1, 2, and 3, while it remained white in setup 4. The Raman peak at 326 cm^−1^, indicative of crystal structure changes, was observed in setups 1, 2, and 3 but not in 4. To isolate the effects of long-living active species, we also conducted experiments by introducing potassium azide into plasma-treated liquid nitrogen, which resulted in no observable changes in color, crystal structure, or polymerization. New IR peaks were only detected in the direct treatment of the powder with plasma (their intensity decreased with distance), indicating the importance of the plasma-produced reactive species.

Our findings suggest that plasma-produced UV radiation is the main factor contributing to the observed color and crystal structure changes in potassium azide following plasma treatment. Its polymerization, on the other hand, was only detected with unobstructed contact with the discharge, suggesting that the main contributors are plasma-produced reactive species. Liquid nitrogen acts not only as a quenching agent that helps to stabilize the product but also as a starting material for the formation of the polynitrogen material. Whether the crystal structure change is also a prerequisite or a byproduct of polymerization remains unclear. Therefore, further research is necessary to elucidate the detailed mechanisms underlying the polymerization process.

## 4. Conclusions

In this study, we report a basic characterization of potassium azide treated via nanosecond-pulsed spark discharge in liquid nitrogen. The optical characterizations conducted for the treatment of potassium azide via nanosecond-pulsed spark discharge support the hypothesis that, in liquid nitrogen, nanosecond-pulsed spark discharge induces significant crystal and structural transformations in potassium azide. These transformations may lead to the polymerization of nitrogen, resulting in a p6/mmm crystal structure with an N_6_ planar ring located at the unit cell’s corner. The observed color and crystal structure changes in potassium azide were attributed to the effects of plasma-produced UV radiation, while the polymerization mechanism is believed to be driven by the active species generated within the discharge.

## Figures and Tables

**Table 1:** List of calculated IR vibrational frequencies of a D2 hexaazabenzene molecule [28] compared with the observed frequencies.

Observed IR Frequency, cm^−1^	Symmetry	Prediction Method					
		PW91		B3LYP		CCSD(T)	
		Frequency, cm^−1^	Intensity	Frequency, cm^−1^	Intensity	Frequency, cm^−1^	Intensity
555	B3	588.5	11.4				
705	B2	710.4	9.7	735.3	6.8	724	7.2
840	B3	872.3	33.6	858.8	9.2	850.1	8.4
1008	B1	1002.8	12.3				
1184	B1	1144.1	29.2	1150	2.1		
	B3	1184.3	24.1				
1384	B2			1367.7	2.8	1330.5	2.5

**Table 2:** Experimental information for the mechanism studied.

Setup	Filter Material Used	Factors Eliminated	Factors in Function	Color Change Observed	Crystal Structure Change	Polymerization
0	N/A		Active species, electric field, UV, visible light, IR	Yes	Yes	Yes
1	Quartz	Active species	UV, electric field, visible light, IR	Yes	Yes	No
2	Metal mesh	Electric field	Active species, UV, visible light, IR	Yes	Yes	No
3	Quartz and metal mesh	Active species, electric field	UV, visible light, IR	Yes	Yes	No
4	Glass	Active species, UV	Electric field, visible light, IR	No	No	No

## Data Availability

The original contributions presented in the study are included in the article, further inquiries can be directed to the corresponding author.
